# Whole genome sequence based capsular typing and antimicrobial resistance prediction of Group B streptococcal isolates from colonized pregnant women in Nigeria

**DOI:** 10.1186/s12864-021-07929-z

**Published:** 2021-08-23

**Authors:** Mienye Bob-Manuel, Lesley McGee, Jeremiah A Igunma, Mary A Alex-Wele, Orikomaba K Obunge, Kennedy T Wariso

**Affiliations:** 1 Department of Medical Microbiology and Parasitology, Rivers State University Teaching Hospital, Port Harcourt, Nigeria; 2 Respiratory Diseases Branch, Centers for Disease Control and Prevention, 1600 Clifton Rd, GA 30329 Atlanta, USA; 3 Department of Medical Microbiology, University of Benin Teaching Hospital, Benin City, Nigeria; 4 Department of Medical Microbiology and Parasitology, University of Port Harcourt Teaching Hospital, Port Harcourt, Nigeria

**Keywords:** Whole genome sequencing, Group B Streptococcus, Nigeria, capsular typing, Antimicrobial resistance

## Abstract

**Background:**

*Streptococcus agalactiae* (Group B Streptococcus, GBS) is one of the major bacterial pathogens responsible for neonatal sepsis. Whole genome sequencing has, in recent years, emerged as a reliable tool for capsular typing and antimicrobial resistance prediction. This study characterized vaginal and rectal isolates of Group B Streptococcus obtained from pregnant women in Port Harcourt, Nigeria using a whole-genome sequence-based approach.

**Results:**

Capsular types Ia, Ib, II, III, IV and V were detected among the 43 isolates sequenced. Twelve sequence types (STs) were identified, with ST19 (*n* = 9, 27.3 %) and ST486 (*n* = 5, 15.2 %) the most frequent among non-duplicated isolates. Of the alpha-like proteins (alp) identified, Alp1 was the most prevalent in 11 (33.3 %) isolates. Macrolide and lincosamide resistance determinants were present in 15 (45.5 %) isolates; *ermB* was detected in 1 (3 %), *ermTR* in 7 (21.2 %) isolates, *lnu* gene was detected in 6 (18.2 %) and *mef* was identified in 3 (9.1 %) isolates. Resistance of GBS to erythromycin and clindamycin (predicted from presence of *erm* or *mef* genes) was found to be 30.3 % and 24.2 %, respectively. All isolates were predicted resistant to tetracycline with only the *tetM* gene identified. Fluoroquinolone-resistance conferring substitutions in *gyrA* + *par*C were detected in 9 (27.3 %) isolates and chloramphenicol resistance was predicted from presence of *aac6’-aph2* gene in 11 (33.3 %).

**Conclusions:**

The data available from the whole genome sequencing of these isolates offers a small but insightful description of common serotypes and resistance features within colonizing GBS in Nigeria.

**Supplementary Information:**

The online version contains supplementary material available at 10.1186/s12864-021-07929-z.

## Introduction

*Streptococcus agalactiae*, also known as Group B Streptococcus (GBS) is increasingly being recognized in Africa as one of the major pathogens responsible for neonatal sepsis [1–3]. Vertical transmission from a rectovaginally colonized mother is the main predisposing factor for the development of early onset neonatal GBS sepsis [4, 5]. Some countries, including the United States and Australia, have policies regarding intrapartum antibiotic prophylaxis (IAP) to prevent early-onset disease (EOD) with resulting benefits [6, 7]. However, many low and middle income countries are yet to develop or implement such policies [8]. Whilst vaccine development is still underway [9, 10], IAP remains one of the most effective tools for the prevention of EOD [7, 11]. The use of IAP may inadvertently result in the increase of antimicrobial resistance (AMR) [8]. For this reason, determination of antimicrobial resistance patterns is important, especially in settings such as Nigeria where there is high incidence of indiscriminate antibiotic use [12]. Whole genome sequencing (WGS) has emerged as a reliable and cost-effective method for capsular typing and for accurate predictions of minimum inhibitory concentrations for antimicrobials in GBS [13–15].

This study analysed whole genome sequences of colonizing Group B Streptococcus isolates from vaginal and rectal swabs obtained from a previously described study in pregnant women in Port Harcourt, Nigeria [16]. Data from this study will add to existing evidence to guide vaccine efforts as well as contribute to local recommendations for intrapartum antibiotic prophylaxis.

## Materials and methods

### Bacterial isolates

A total of 46 GBS isolates, obtained from separate vaginal and rectal samples of 185 pregnant women in their third trimester, collected retrospectively between March and July 2018 were used in this study. Samples were collected from women receiving antenatal care at the University of Port Harcourt Teaching Hospital in Port Harcourt Nigeria. Ethical approval was received from the hospital’s ethics board and each participant signed a consent form prior to enrolment. Details of the study design and sample collection are described previously by Bob-Manuel et al. [16]. Briefly, the rectovaginal samples were incubated overnight in LIM RambaQUICK™ (CHROMagar, France) and subcultured onto CHROMagar™ StrepB (CHROMagar, France). *Streptococcus agalactiae* were presumptively identified as small, mauve-coloured colonies on CHROMagar StrepB. Isolates were stored in tryptic soy broth containing 5 % glycerol at -70 °C. The isolates were shipped on Columbia agar with chocolated horse blood (Oxoid, UK) to the Streptococcus Laboratory, Centers for Disease Control and Prevention (CDC), for whole genome sequencing.

### Whole genome sequencing and strain feature prediction

At CDC, GBS isolates were cultured on Trypticase soy agar supplemented with 5 % sheep blood prior to DNA extraction from single colonies as previously described [13]. Following shearing of samples using a Covaris M220 ultrasonicator (Covaris, Inc, MA, USA) genomic libraries were constructed using sparQ DNA Library Kit (Quantabio, MA USA) [13, 14]. Short read sequences were generated on Illumina Miseq platform with MiSeq v2 500 cycle kit (Illumina Inc, USA) and assembled de novo using VelvetOptimiser [17]. Serotypes, multilocus sequence typing (MLST), antibiotic resistance determinants, and predicted minimum inhibitory concentrations (MICs) were determined using a previously validated GBS bioinformatics pipeline [13]. Isolates flagged for phenotypic testing (those with *lnu* gene and *erm*TR + *lnu*) were tested by Etest (Biomerieux USA) using Mueller-Hinton agar with 5 % sheep blood (Becton Dickinson, USA), according to manufacturer’s instructions. Interpretation of susceptible, intermediate and resistant from MIC WGS-prediction and Etest were determined using the standard Clinical Laboratory Standards Institute (CLSI) breakpoints for beta-hemolytic streptococci [18]. Additional strain features, including surface protein genes encoding the hypervirulent GBS adhesin (*hvga*), serine-rich repeat (*srr*) proteins, alpha protein family (alpha, Rib, Alp2/3, Alp1), and pilus proteins predictive of pilus islands (designated here as PI1, PI2A, and PI2B) were extracted from genomic data (https://github.com/BenJamesMetcalf) [14]. Isolate identifiers, pipeline features, antimicrobial MIC predictions and interpretations, assembly metrics and NCBI genome accession numbers are listed in Supplementary Table 1. Contingency tables and a *χ*^2^ test (or Fisher’s exact test) were used to determine the significance of associations (at *α* = 0.05).

## Results

The proportion of vaginal/rectal samples positive for GBS was 16.8 % (31/185 women) (sTable 1); 12 women had both vaginal and rectal swabs positive for GBS with 10 women having identical serotypes and therefore counted only once in further analyses. Whole genome sequencing revealed 3 of the isolates to be non-GBS. Six of ten known capsular serotypes (Ia, Ib, II, III, IV and V) were detected within this sampling, with serotype V the most frequent (*n* = 11/33, 33.3 %) (Table 1). Multilocus sequence typing (MLST) identified a total of 12 STs among the 33 strains with ST19 (*n* = 9, 27.3 %) and ST486 (*n* = 5, 15.2 %) most frequently occurring. Eight (88.9 %) of the ST19 sequence strains were serotype V isolates while serotype III accounted for 1 (11.1 %) isolate. Only 1 serotype III isolate (3 %) had the highly virulent ST17 clonal type.

**Table 1:** Distribution of sequence types (ST) by serotype for 33 non-duplicate GBS isolates

ST	Serotypes (%)					
	**Ia**	**Ib**	**II**	**III**	**IV**	**V**
1	-	-	-	-	-	1 (9.1)
2	-	-	-	-	2 (100.0)	-
8	-	1 (100)	-	-	-	-
17	-	-	-	1 (14.3)	-	-
19	-	-	-	1 (14.3)	-	8 (72.7)
23	1 (16.7)	-	-	-	-	-
24	4 (66.7)	-	-	-	-	-
26	-	-	-	-	-	1 (9.1)
28	-	-	2 (33.3)	-	-	1 (9.1)
182	-	-	-	3 (42.9)	-	-
486	1 (16.7)	-	4 (66.7)	-	-	-
1336	-	-	-	2 (28.6)	-	-
**Total**	**6 (18.2)**	**1 (3.0)**	**6 (18.2)**	**7 (21.2)**	**2 (6.1)**	**11 (33.3)**

The most predominant alpha-protein-like (alp) family present was Alp1 in 11 (33.3 %) isolates, followed by Rib with 9 (27.3 %), Alp2/3 with 6 (18.23 %) isolates and 5 (15.2 %) isolates were Alpha. Rib was detected in capsular types III (85.7 %) (*p* < 0.001), II (33.3 %) and V (9.1 %); alp2/3 was detected in II (66.7 %) (*p* = 0.002), Ia (16.7 %) and V (9.1 %); alp 1 in Ia (16.7 %), IV (100 %) and V (72.7 %) (*p* < 0.001) while the Alpha protein was detected in type Ia (66.7 %) (*p* < 0.001) and Ib (100 %) alone. Two isolates were negative for any of the alpha-protein-like family target predictions.

Serine-rich repeat proteins SSR1 and SSR2 were predicted in 29 (87.9 %) and 1 (3 %) of the isolates, while 3 (9.1 %) were negative for both. Only one isolate demonstrated *hvgA*, a serotype III ST17 isolate as expected, the same isolate that was SSR2 positive. Isolates either had 1 or 2 pili; PI1 + PI2A detected in 19 (57.6 %), PI1 + PI2B in 8 (24.2 %) and PI2A in 6 (18.1 %) isolates. Serotype V isolates mostly contain both PI1 + PI2A (90.9 %) (*p* = 0.003), serotype II had both PI1 + PI2B (66.7 %) (*p* = 0.01) and serotype 1 A, pilus P2A (83.3 %) (*P* < 0.001).

Table 2 shows the antibiotic susceptibility as predicted by WGS and detection of specific resistance determinants. Macrolide and clindamycin resistance determinants (*erm*, *mef*/*msr*D and *lnu*) were present in 15 (45.5 %) of the isolates with resistance rates to erythromycin and clindamycin of 30.3 and 24.2 %, respectively. These determinants were present only in serotypes III (71.4 %), IV (100 %) and V (72.7 %). The MLS_B_ genes which confer resistance to macrolides, lincosamides and streptogramin B, *ermB* was detected in 1 (3.0 %) isolates and *ermTR* in 7 (21.2 %) isolates; lincosamide nucleotidyltransferase determinant, *lnu*, was present in 6 (18.2 %) and the determinant for macrolide efflux, *mef* was detected in 3 (9.1 %) isolates. The 5 (15.2 %) isolates with *lnu* alone and the one isolate with *erm*TR + *lnu* were susceptible to erythromycin + clindamycin and synercid, respectively, when tested phenotypically by Etest. Isolates with *mef/msrD* alone (2; 6.1 %) were predicted as resistant to erythromycin but sensitive to clindamycin.

**Table 2:** Whole Genome Sequencing predicted antibiotic susceptibility for 33 non-duplicate GBS isolates (See Supplemental Table 1 for MIC prediction values)

Antibiotic	Targeted genes	Susceptible (%)	Non-Susceptible *(%)
Ampicillin	*pbp*2X	33 (100)	-
Ceftriaxone	*pbp*2X	33 (100)	-
Chloramphenicol	*cat*	25 (75.6)	8 (24.2)
Clindamycin	*erm*	25 (75.6)	8 (24.2)
Ceftaroline	*pbp*2X	33 (100)	-
Erythromycin	*erm* or *mef*	23 (69.7)	10 (30.3)
Levofloxacin	*par*C, *gyr*A	24 (72.7)	9 (27.3)
Meropenem	*pbp*2X	33 (100)	-
Linezolid	23 S rRNA	33 (100)	-
Penicillin	*pbp*2X	33 (100)	-
Synercid**	*erm* + *lsa*	33 (100)	-
Cefotaxime	*pbp*2X	33 (100)	-
Tetracycline	*tet*	-	33 (100)
Vancomycin	*van*	33 (100)	-

** Quinupristin/Dalfopristin

*Non-susceptibility includes both intermediate and resistant prediction

All isolates were resistant to tetracycline and the only gene detected conferring resistance was *tetM*. Fluoroquinolone-resistance conferring substitutions in both *gyr*A (S81L) and *par*C (S79F) were present in 9 (27.3 %) isolates conferring full resistance phenotype, 8 (88.9 %) of which were ST19 strains (*p* < 0.0001). Also, all of the isolates with fluoroquinolone resistance determinants were serotype V (81.1 %) (*p* < 0.0001). The *aac6’-aph2* gene, which encodes the bifunctional aminoglycoside-inactivating enzyme with 6’-acetyltransferase and 2’’-phosphotransferase activities, were detected in 11 (33.3 %) isolates and were exclusively in serotypes III, IV and V: 4 (36.4 %), 1 (9.1 %) and 6 (54.5 %), respectively. CAT genes, encoding chloramphenicol acetyltransferases, were detected in 8 (24.2 %) isolates, among serotypes III (*p* = 0.02), IV and V.

## Discussion

This study reports the molecular characterization of colonizing strains of Group B Streptococcus in the vagina and rectum of pregnant women. Serotype V was the most frequent serotype in this population representing 33.3 % of the population.

Sequence types ST19, 486, 182, 24 and 2 accounted for 69.7 % of the total isolates. This diversity is different from other reports that have reported ST19, ST23 and ST1 more commonly [19]. The highly virulent ST17 responsible for most invasive disease, especially late onset disease in neonates [20], was rare in this study (3 %) population which may suggest a better prognosis for neonates who may become colonized at birth in this environment. While ST182 was reported as a frequent clonal type by Medugu et al. in another local study in Abuja, Nigeria [2], ST486 has not previously been documented as a common sequence type among GBS isolates in Nigeria. The pubMLST [21] (http://pubmlst.org accessed June 10, 2021) and BacWGSTdb 2.0 [22] (bacdbcn/BacWGSTdb/ accessed June 10th, 2021) show ST182 (Kenya, Malawi and Central African Republic), ST486 (Kenya), ST24 (Kenya and Malawi) and ST2 (Kenya and Ethiopia) from several African countries mainly associated with carriage samples collected during 2006 to 2015, indicating circulation of these less common STs.

Alp1 (Epsilon) (33.3 %) was the most common of all the alpha-like proteins among the isolates in this study, similar to findings reported in Egypt (27 %) [23]. Other studies have documented Rib as the most frequently detected Alp gene in other geographic regions like China and Iran (37.5 and 53 % respectively) [24, 25]. The serine-rich repeat protein Srr-1 was identified in almost all isolates, however only a single isolate had the Srr-2 known to be associated with greater binding affinity and higher morbidity [26]. The same isolate was positive for the hypervirulent GBS adhesin (HvgA), another surface protein demonstrated to increase adhesion and virulence particularly in the ST17 lineage [27].

Similar to other studies from Nigeria [2, 28], all isolates were determined to be susceptible to beta-lactam antibiotics including 3rd generation cephalosporins. This is quite reassuring, suggesting that penicillin and ampicillin remain as viable options for intrapartum antibiotic prophylaxis in this region despite concerns for antimicrobial resistance. For women allergic to penicillin and at high risk of anaphylaxis, guidelines recommend clindamycin as an alternative option for prophylaxis [29]. In this study however, clindamycin and erythromycin resistance were relatively high at 24.2 and 30.3 %, respectively. Data from an earlier study in Nigeria reported erythromycin and clindamycin resistance rates of 6.5 % [28] suggesting increased resistance rates within the country. However, all isolates were susceptible to vancomycin in this study, so patients with a high risk for penicillin anaphylaxis who are colonized with a clindamycin resistant GBS strain, could receive vancomycin as the next best option [29].

Although other antibiotic classes such as fluoroquinolones, chloramphenicol, and tetracycline are not recommended for intrapartum prophylaxis, determining their susceptibility is important for tracking of resistance among GBS in general and as useful options for other invasive group B streptococcal infections in other age groups. All isolates were predicted as tetracycline resistant which is consistent with several other reports documenting high levels of resistance to this antibiotic [28, 30, 31]. Resistance to chloramphenicol and levofloxacin were also high at 25.6 %. Similarly, while these antibiotics may not be recommended for neonatal sepsis, they may be useful options for other invasive group B streptococcal infections in other age groups.

The only tetracycline gene present in this population was *tet*M. Of all the tetracycline resistance determinants, the *tet*M has the most spread geographically among GBS [28, 30, 31]. The presence of the double *gyr*A + *par*C mutants conferring full fluoroquinolone-resistance was high and were found predominantly in serotype V/ST-19 clone. The most frequently reported fluoroquinolone non-susceptible serotype/ST combinations are III/ST-19 and V/ST-19 [32, 33] supporting the data from this study. Occurrence of either of these clonal complexes is likely a reflection of the circulating clones in those geographical areas in which they are found.

This study has demonstrated some salient characteristics of the prevalent colonizing strains of GBS in Nigeria, thus, contributing to the body of knowledge concerning GBS, as well as providing AMR data if IAP is considered in the future.

## Supplementary information

**Additional file 1.**

## Data Availability

Genome sequences has been uploaded to GenBank https://www.ncbi.nlm.nih.gov/sra/PRJNA632041. See sTable 1 for BioSample Accession numbers (SAMN18352391- SAMN18352411).
